# Caracterización molecular y fenotípica de aislamientos clínicos de *Salmonella* Typhimurium variante monofásica (1,4,[5],12:i:-) recuperados en Colombia

**DOI:** 10.7705/biomedica.5417

**Published:** 2020-12-11

**Authors:** Paloma Cuenca-Arias, Lucy Angeline Montaño, José Miguel Villarreal, Magdalena Wiesner

**Affiliations:** 1 Grupo de Microbiología, Subdirección de Investigación Científica y Tecnológica, Dirección de Investigación en Salud Pública, Instituto Nacional de Salud, Bogotá, D.C., Colombia Grupo de Microbiología Instituto Nacional de Salud BogotáD.C Colombia; 2 Grupo de Microbiología, Dirección de Redes en Salud Pública, Instituto Nacional de Salud, Bogotá, D. C., Colombia Grupo de Microbiología Instituto Nacional de Salud Bogotá Colombia; 3 Departamento de Química, Facultad de Ciencias, Universidad Nacional de Colombia, Bogotá, D. C., Colombia Universidad Nacional de Colombia Departamento de Química Facultad de Ciencias Universidad Nacional de Colombia BogotáD. C Colombia; 4 Grupo de Investigación en Ciencias Biológicas y Químicas, Facultad de Ciencias, Universidad Antonio Nariño, Bogotá, D. C., Colombia Universidad Antonio Nariño Grupo de Investigación en Ciencias Biológicas y Químicas Facultad de Ciencias Universidad Antonio Nariño BogotáD. C Colombia

**Keywords:** *Salmonella* Typhimurium, porinas, flagelos, vigilancia, Colombia, *Salmonella* Typhimurium, porins, flagella, surveillance, Colombia

## Abstract

**Introducción.:**

La variante monofásica (1,4,[5],12:i:-) de *Salmonella* Typhimurium ocupa los primeros lugares en los programas de vigilancia de *Salmonella* a nivel mundial. En Colombia, *Salmonella enterica* variante monofásica alcanza el cuarto lugar en cuanto a los aislamientos clínicos recuperados por medio de la vigilancia por laboratorio del Grupo de Microbiología del Instituto Nacional de Salud, pero se desconoce si dichos aislamientos están relacionados con la variante monofásica de Typhimurium que circula a nivel global, y con sus características genéticas y fenotípicas.

**Objetivo.:**

Caracterizar los aislamientos de *Salmonella* monofásica recuperados en Colombia entre el 2015 y el 2018 por el Grupo de Microbiología del Instituto Nacional de Salud.

**Materiales y métodos.:**

Se analizaron 286 aislamientos clínicos de *Salmonella enterica* variante monofásica mediante PCR o secuenciación del genoma completo *(Whole Genome Sequencing,* WGS) para confirmar si correspondían a *Salmonella* Typhimurium variante monofásica, en tanto que, en 54 aislamientos, se determinó la estructura genética del operón que codifica la segunda fase flagelar y, en 23, se evaluó la motilidad, el crecimiento y la expresión de las proteínas de membrana externa.

**Resultados.:**

El 61 % (n=174) de los aislamientos de *Salmonella* monofásica correspondió a *Salmonella* Typhimurium serovar monofásico. El 64,8 % (n=35/54) se relacionó con el clon europeo-español y, el 13 % (n=7/54), con el estadounidense. En dos aislamientos de orina se encontró una diferencia significativa en la motilidad y el crecimiento, así como ausencia de la porina OmpD en medio mínimo M9.

**Conclusiones.:**

En el periodo de estudio, circuló en Colombia la variante monofásica de *Salmonella* Typhimurium relacionada con el clon europeo-español, y se registró ausencia total del operón *fljAB.* Los resultados evidenciaron cambios fenotípicos en los aislamientos provenientes de muestras de orina que sugieren adaptación en procesos invasivos.

## Abstract (en)

**Introduction.:**

The *Salmonella* Typhimurium monophasic variant (1,4,[5],12:i:-) is currently the most commonly detected variant in *Salmonella* surveillance programs worldwide. In Colombia, the *Salmonella enterica* monophasic variant is the fourth most common clinical isolate recovered through the laboratory surveillance of the *Grupo de Microbiología* from the *Instituto Nacional de Salud;* however, it is unknown whether these isolates are closely related to the monophasic Typhimurium variant, which circulates globally, and their genetic and phenotypic characteristics have not been reported.

**Objective.:**

To characterize monophasic *Salmonella enterica* isolates identified in Colombia from 2015 to 2018 by the *Instituto Nacional de Salud.*

**Materials and methods.:**

Two hundred eighty-six clinical isolates of the monophasic *Salmonella enterica* variant were analyzed by PCR or whole-genome sequencing to confirm whether they corresponded to the *Salmonella* Typhimurium monophasic variant while the genetic structure of the operon encoding the second flagellar phase was determined in 54 isolates. Motility, growth, and expression of the outer membrane proteins were evaluated in 23 isolates.

**Results.:**

During the study period in Colombia, 61% (n=174) of *Salmonella* monophasic isolates belonged to *Salmonella* Typhimurium serovar monophasic (1,4,[5],12:i-). Of these, 64.8% (n=35/54) were related to the European/Spanish clone and 13% (n=7/54) to the U.S. clone. Two isolates recovered from urine samples showed differences in motility, growth, and the absence of the OmpD porin in M9 minimal medium.

**Conclusions.:**

Most of the monophasic *Salmonella* Typhimurium variants that have circulated in Colombia since 2015 lacked the second phase of operon *fljAB,* which is related to the European/Spanish clone. The results evidenced phenotypic changes in urine samples suggesting bacterial adaptation in the case of these invasive samples.

*Salmonella* spp. es una de las principales causas de enfermedad diarreica a nivel global; según la Organización Mundial de la Salud (OMS), una de cada diez personas adquiere este agente patógeno por el consumo de agua o comida contaminadas y anualmente se reportan más de 550 millones de casos ^1^.

La clasificación de *Salmonella* spp. se hace con el método de serotipificación siguiendo el esquema de Kauffmann-White-Le Minor, que identifica los antígenos presentes en la superficie bacteriana, como el lipopolisacárido (antígeno somático O), las proteínas flagelares (antígeno H) y las capsulares (antígeno K) ^2^. La mayoría de los serovares de *Salmonella* spp. son móviles debido a las proteínas flagelares que están codificadas por dos genes cromosómicos diferentes, el *fliC* para la primera fase y el *fljB* para la segunda, los cuales se expresan de manera alternada mediante el mecanismo de variación de fase flagelar. La responsable de este mecanismo es la unidad genética del operón fljAB, compuesta por la enzima ADN invertasa hin, que actúa como un interruptor molecular, seguida del gen *fljA,* que codifica un regulador negativo inhibidor de la expresión de la primera fase flagelar *(fliC),* y el gen *fljB,* que expresa la proteína flagelar de la fase dos ^3^^,^^4^. Estos serovares son bifásicos, es decir, son capaces de expresar ambos genes flagelares.

*Salmonella enterica,* subespecie *enterica* serovar Typhimurium (Typhimurium), es el principal serotipo a nivel mundial proveniente de muestras clínicas. La serotipificación de este serovar bifásico incluye el reconocimiento de las dos fases flagelares y su fórmula antigénica 1,4,[5],12:i:1,2. Sin embargo, en Europa y Estados Unidos, la variante de Typhimurium monofásica-STVM (1,[4],5,12:i:-), que se asocia con multirresistencia y no expresa la segunda fase flagelar, se cuenta entre las más frecuentemente recuperadas en los aislamientos clínicos ^5^^,^^6^. Dado que en la serotipificación solo se identifican las proteínas expresadas, estos aislamientos suelen clasificarse inicialmente como S. *enterica* subsp. *enterica* serovar (1,4,[5],12:i:-), o serovar monofásico, por lo que la confirmación de la variante STVM puede hacerse únicamente mediante técnicas moleculares como la PCR o la secuenciación de genoma completo.

En los informes internacionales, y mediante análisis filogenéticos, se han identificado varios clones de STVM con diferentes mecanismos de resistencia, perfiles de electroforesis en gel de campo pulsado (PFGE) y patrones de análisis de repetición en tándem de un número variable de múltiples locus ^7^^-^^9^, que se caracterizan por la pérdida de regiones genéticas a lo largo del cromosoma bacteriano o por la adquisición de elementos de resistencia a antibióticos y metales pesados, lo que sugiere la aparición de la STVM por medio de eventos independientes ^10^. Entre ellos, se destacan tres clones descritos por Soyer, *et al.*^11^: el clon europeo y el clon español, que son multirresistentes y carecen por completo del operón *fljAB,* pero difieren en el tipo de "secuenciotipo" ST34 y ST19, la ausencia y la presencia del gen *iroB* y el plásmido de virulencia de *Salmonella,* respectivamente, y el clon estadounidense, el cual es sensible a los antibióticos y conserva los genes *hin* e *iroB.*

Actualmente, el clon europeo es el predominante a nivel global. Todos los clones presentan grandes deleciones en el contenido del genoma. Además de estos clones reportados a nivel internacional, se han descrito clones endémicos en países como Bélgica, Japón y Estados Unidos, los cuales exhiben otros arreglos de genes en el operón *fljAB*^11^^,^^12^. En Colombia, predomina el serovar Typhimurium según los resultados obtenidos por el Programa de Vigilancia por el Laboratorio de la Enfermedad Diarreica Aguda del Grupo de Microbiología del Instituto Nacional de Salud ^13^^,^^14^ utilizando el esquema de Kauffmann-White-Le Minor ^2^.

Entre el 2015 y el 2017, el serovar identificado como *Salmonella enterica* subsp. *enterica* serovar (1,4,[5],12:i:-) se ubicó en el cuarto lugar de la vigilancia, con 180 aislamientos del total de 12.055. Mediante la caracterización genómica de 209 aislamientos colombianos de Typhimurium recuperados de hemocultivos, se confirmó que 16 de ellos, recuperados entre el 2015 y el 2016 y clasificados como serovar monofásico (1,4,[5],12:i:-), pertenecían a la variante STVM ^15^, aunque solo representaron, aproximadamente, el 9 % de los aislamientos monofásicos recuperados en el país.

Dado el rápido y reciente incremento de este serovar a nivel mundial y local, es importante confirmar si el total de aislamientos colombianos pertenecen a la variante STVM, con el fin de estar alerta ante un posible reemplazo de serovar. El objetivo del presente estudio fue confirmar si los aislamientos de S. *enterica* subsp. *enterica* serovar (1,4,[5],12:i:-) recuperados en el país durante el periodo de estudio correspondían a la STVM y a los clones ampliamente distribuidos, utilizando las pruebas de PCR, secuenciación de genoma completo *(Whole Genome Sequencing,* WGS), el fenotipo de crecimiento y los ensayos de motilidad.

## Materiales y métodos

### Aislamientos clínicos

Se analizaron 286 aislamientos clínicos de S. *enterica* subsp. *enterica* serovar (1,4,[5],12:i:-) recuperados de muestras de materia fecal (n=133), hemocultivo (n=105), orina (n=19), otras muestras (n=18) y aquellas sin datos (n=11) entre el 2015 y el 2018 por medio de la vigilancia por el laboratorio de la enfermedad diarreica aguda que se lleva a cabo en el Instituto Nacional de Salud (cuadro 1).

**Cuadro 1:** Total de aislamientos de *Salmonella* Typhimurium variante monofásica (1,4,[5],12:i:-), aislamientos positivos para el serovar STVM y porcentaje por año en Colombia

Año	n	**Positivas *fliAB***	(%)
2015	58	41	70,7
2016	72	38	52,8
2017	52	34	65,4
2018	104	61	58,7
Total	286	174	60,8

La serotipificación se realizó siguiendo el esquema de Kauffman-White-Le Minor ^2^. La sensibilidad antimicrobiana se evaluó frente a ampicilina, ceftazidima, trimetoprim-sulfametoxazol, cefotaxima, cloranfenicol, tetraciclina y ácido nalidíxico mediante la técnica de difusión en disco de Kirby-Bauer y métodos semiautomatizados VITEK™, siguiendo las guías del *Clinical and Laboratory Standards Intitute* (CLSI) ^16^.

Para las pruebas de PCR, el ADN total se extrajo con el método de ebullición en 272 aislamientos ^17^. En los restantes 14 aislamientos, se secuenció genoma completo como parte del proyecto "10,000 *Salmonella* Genomes" mediante extracción con el paquete MagAttract™ (Qiagen), y la secuenciación con Illumina HiSeq4000™. Los números de acceso *(Sequence Read Archive Accession Numbers)* de los aislamientos se presentan en el cuadro 2 ^15^^,^^18^. Se utilizó Typhimurium ATCC 14028 como cepa de referencia ^19^.

**Cuadro 2:** Aislamientos de *Salmonella* Typhimurium variante monofásica-STVM (1,4,[5],12:i:-) (n=23) seleccionados para curvas de crecimiento, pruebas de motilidad y expresión de OMP

Código	Año	Muestra	Resistencia	Linaje clonal	Número de acceso SRA
1	2015	Materia fecal	TET	Inconsistente	No aplica.
2	2015	Materia fecal	TET	Variante atípica	No aplica.
3	2016	Hemocultivo	TET, CHL, NAL	Variante atípica	No aplica.
4	2016	Materia fecal	TET	Europeo-español	No aplica.
5	2016	Materia fecal	TET, CHL, SXT, AMP	Europeo-español	No aplica.
6	2017	Materia fecal	TET, CHL, AMP	Europeo-español	No aplica.
7	2017	Orina	Sensible	Europeo-español	No aplica.
8	2018	Hemocultivo	CTX, CAZ	Estados Unidos	No aplica.
9	2018	Orina	TET, NAL, AMP	Estados Unidos	No aplica.
10	2015	Hemocultivo	TET, CHL, NAL	Europeo-español	SRR8740456
11	2015	Hemocultivo	TET, CHL, NAL	Europeo-español	SRR8740455
12	2015	Hemocultivo	TET, CHL, NAL	Europeo-español	SRR8740452
13	2015	Hemocultivo	TET, CHL, NAL	Europeo-español	SRR8740488
14	2015	Hemocultivo	TET, NAL	Europeo-español	SRR8740487
15	2016	Hemocultivo	TET	Europeo-español	SRR8740503
16	2016	Hemocultivo	TET, CHL	Estados Unidos	SRR8740534
17	2016	Hemocultivo	TET	Europeo-español	No aplica.
18	2016	Hemocultivo	TET, CHL, AMP	Estados Unidos	SRR8740536
19	2016	Hemocultivo	TET, CHL	Europeo-español	No aplica.
20	2016	Hemocultivo	TET, CHL	Europeo-español	No aplica.
21	2016	Hemocultivo	Sensible	Estados Unidos	SRR8740431
22	2016	Hemocultivo	TET, AMP	Estados Unidos	SRR8740430
23	2016	Hemocultivo	TET, AMP	Europeo-español	SRR8740429

SRA: *Sequence Read Archive ;* CHL: cloranfenicol; NAL: ácido nalidíxico; TET: tetraciclina; CAZ: ceftazidima; AMP ampicilina; STX: trimetoprim-sulfametoxazol

### Caracterización molecular

Para determinar cuáles de los aislamientos identificados como S. *enterica* subsp. *enterica* serovar (1,4,[5],12:i:-) correspondían a la STVM, se utilizó la PCR descrita por Echeita, *et al.*^20^, la cual se basa en la amplificación de la región intergénica de los genes del operón que codifica para la primera fase flagelar *fliB-fliA.*

Los aislamientos de Typhimurium específicamente tienen una inserción de un fragmento IS200 en esta región intergénica, lo que resulta en la amplificación de una banda de 1.000 pb, en tanto que la amplificación de esta región para los otros serovares de *Salmonella* genera un fragmento de 250 pb ^20^ (cuadro 3).

**Cuadro 3:** Oligonucleótidos utilizados en este estudio

Blanco genético	Oligonucleótidos	Secuencia 5’-3’	Tamaño de amplicón (pb)	Referencia
Región entre *fliA* y *fliB*	*fliA-fliB*-F	ctg gcg acg atc tgt cga tg	1.000	^20^
	*fliA-fliB*-R	gcg gta tac agt gaa ttc ac		
*fljA*	*fljA*-F	ttc att agg tcc cct ccg g	1.049	^11^
	*fljAB*-R	att cag ccc cgt gaa ttc ggg		
*fljB*	*fljBH*-F	ttt acc gtc tac gcc acc c	551	^11^
	*fljBH*-R	ggt act aca ctg gat gta tcg g		
*hin*	*hinF*-F	tgg cta cta ttg ggt ata ttc ggg	473	^11^
	*hinF*-R	aat tca ttc gtt ttt tta tgc ggc		

La secuencia hacia adelante se indica con "F" y la secuencia inversa con "R'.'

**Figura 1:**
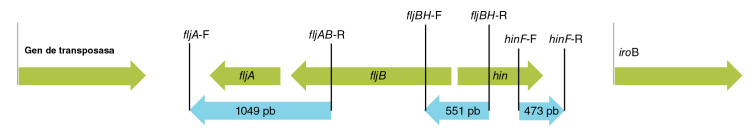
Organización genética del operón *fljAB* en Typhimurium. El tamaño de amplificación (pb) esperado para los genes del operón se encuentra señalado por flechas de color azul flanqueadas por el nombre de los respectivos oligonucléotidos.

Se seleccionaron 54 aislamientos de los confirmados como STVM (cuadro 4) para evaluar la presencia de los genes del operón *fljAB* mediante tres juegos de iniciadores que amplifican las regiones intergénicas comprendidas entre los tres genes del operón (cuadro 3), según lo descrito por Soyer, et *al.*^11^, como se observa en la figura 1. En los 14 aislamientos estudiados con secuenciación de genoma completo, los genes del operón *fljAB* se analizaron *in silico* usando la herramienta bioinformàtica del *Pathosystems Resource Integration Center* (PATRIO: https://www.patricbrc.org/) ^21^.

**Cuadro 4:** Identificación de los genes del operón *f¡AB* mediante PCR en 54 aislamientos confirmados como STVM. Se muestran las configuraciones de los genes con relación a los clones descritos y el tipo de muestra clínica del cual fueron recuperados.

	**Estructura del operón *fljAB***					Muestra		
Linaje clonal	*fljA*	*fljB*	*hin*	n (%)	Materia fecal	Hemocultivo	Orina	Otras muestras
Europeo-español	-	-	-	35 (64,8)	16	15	1	3
Estados Unidos	-	-	+	7(13)	1	5	1	0
Endémico	-	+	+	7(13)	4	2	0	1
Inconsistente	+	+	+	3 (5,6)	3	0	0	0
Variante atípica	-	+	-	2 (3,7)	1	1	0	0
		Total		54	25	23	2	4

### Análisis estadístico

Mediante la prueba de ji al cuadrado (estadísticamente significativo: p<0,05), se determinó la relación entre el tipo de muestra (hemocultivo o materia fecal) en el que se recuperó el aislamiento STVM y la identificación del clon europeo-español por ser el predominante.

### Caracterización fenotípica

De los 54 aislamientos de STVM caracterizados, se seleccionaron 23 representativos de diferentes tipos de muestra y clones para evaluar la curva de crecimiento y hacer las pruebas de motilidad (cuadro 2). Se utilizó el medio Luria Bertani para imitar las condiciones del intestino delgado, donde la bacteria tiene todos los nutrientes a su disposición y una alta osmolaridad ^17^.

Para simular las condiciones dentro del macròfago, se preparó el medio mínimo M9 con glucosa al 20 % como única fuente de carbono ^17^. Se emplearon los medios nutritivos Luria Bertani y mínimo M9 para las curvas de crecimiento en medio líquido a las 24 horas y las pruebas de motilidad en agar blando al 0,3 % a las 8 y las 24 horas, hechas por triplicado siguiendo a Bogomolnaya, *et al.*^22^. Las proteínas de membrana externa *(Outer Membrane Proteins,* OMP) se obtuvieron de los aislamientos de STVM sembrados en los medios mínimo M9 y nutritivo Luria Bertani hasta una DO_600_ de 0,6 y se corrieron en SDS-PAGE con acrilamida/bisacrilamida al 10 %, según lo descrito por Villarreal, *et al.*^23^.

## Resultados

### En Colombia circulan aislamientos de Typhimurium de la variante monofásica (STVM).

El 61 % (n=174) de los 286 aislamientos serotipificados como *Salmonella entérica* subsp. *entérica* serovar (1,4,[5],12:¡:-) entre el 2015 y el 2018, correspondieron a STVM (cuadro 1); el 39 % (n=112) restante, sin expresión de la segunda fase flagelar, podrían estar relacionados con otros serotipos de fórmula antigénica similar a Typhimurium como el serovar Lagos (4,[5],12:i:1,5), el serovar Agama (4,12:i:1,6), el serovar Tsevie (4,12:i:e,n,z_16_), y el serovar Tumodi (1,4,12:i:z_6_) (11), aunque esto no se confirmó.

De los 174 aislamientos confirmados como STVM, 81 (46,5 %) provenían de muestras de materia fecal, 66 (38 %) de hemocultivo, 11 (6,3 %) de orina, 9 (5,2 %) de otras muestras y 7 (4 %) no contaban con información registrada.

**Figura 2:**
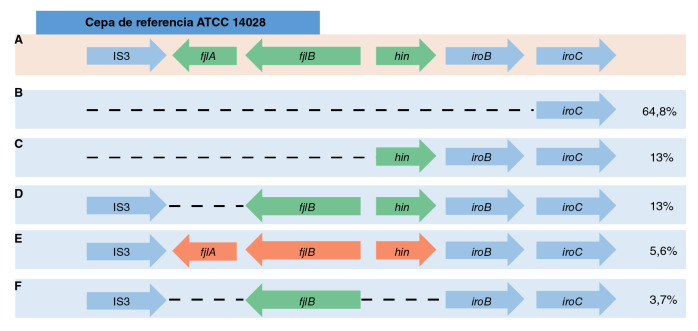
Esquema que representa los patrones de deleción de genes en el operón *fljAB*. A) Estructura del operón *fljAB* de la cepa de referencia Typhimurium ATCC 14028. B) Deleción completa del operón *fljAB* en el clon europeoespañol. C) Arreglo genético del clon estadounidense. D) Arreglo genético de la variante endémica atípica que conserva el gen *fljB* y el gen *hin*. E) Variante inconsistente que conserva el operón *fljAB* completo. F) Variante atípica que conserva el gen *fljB*. Se indica el porcentaje de cada linaje derivado del cuadro 3.

Se analizó la estructura del operón *fljAB* en 54 de ellos mediante PCR y secuenciación de genoma completo (cuadro 4) y se encontró que el 64,8 % (n=35) presentaba una deleción total del operón, similar a la reportada en el clon europeo-español ^24^. Siete (13 %) aislamientos perdieron la parte inicial del operón (ausencia del gen *fljA)* y conservaron los otros dos genes *fljB-hin;* estas modificaciones, que no son muy comunes, se identificaron como clones endémicos. De estos, el 5,6 % (n=3) portaba el operón completo y el restante 3,7 % (n=2) correspondía a las denominadas variantes monofásicas "atípicas" de Typhimurium, según lo descrito por Hopkins, et *al.*^25^, las cuales portan el gen que codifica para la proteína flagelar de segunda fase *(fljB),* pero no la expresan al no poseer el promotor del operón *fljAB* ni el represor de la proteína FliC (cuadro 4, figura 2).

No se observó correlación estadística entre el tipo de muestra del aislamiento (hemocultivo, materia fecal) y el clon europeo-español (p=0,910; OR=0,937) (cuadro 4).

En los 23 aislamientos seleccionados para las pruebas de curva de crecimiento y motilidad, se observó resistencia a tetraciclina en el 87 % (20/23), a cloranfenicol en el 478 %, a ácido nalidíxico en el 30,4 % y a ampicilina en el 26 %. Nueve aislamientos fueron resistentes a tres antimicrobianos y la combinación predominante fue la de tetraciclina, cloranfenicol y ácido nalidíxico en el 21,7 % de ellos (cuadro 2).

El crecimiento bacteriano de estos aislamientos, evaluado tanto en medio nutritivo Luria Bertani como en medio mínimo M9, no se vio afectado en comparación con la cepa de referencia ATCC 14028 (no se presentan los datos), con excepción de los aislamientos provenientes de muestras de orina (denominados 7 y 9), los cuales alcanzaron una DO a 600 nm de 0,136 y 0,803, respectivamente, después de 24 horas, lo que sugiere que hubo inhibición del crecimiento en el medio mínimo.

**Figura 3:**
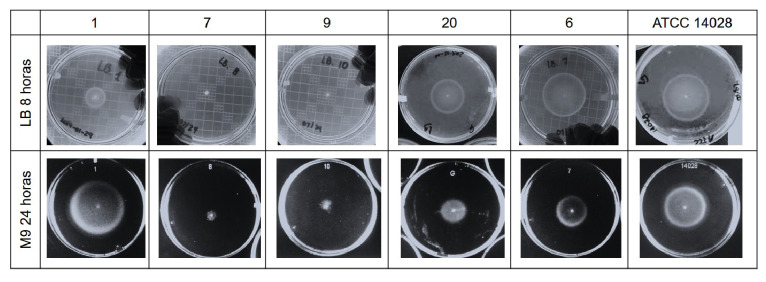
Evaluación de la motilidad en aislamientos clínicos de STVM. La motilidad se evaluó a las 8 y 24 horas de incubación a 37 °C en placas con agar blando (0,3 %) y suplemento de LB (fila superior) o medio mínimo M9 (fila inferior). En la figura se muestran los aislamientos 1, 7, 9, 20, los cuales registraron diferencias en la motilidad con respecto a la cepa de control ATCC 14028, y el aislamiento 6 a manera de comparación general del comportamiento de los restantes 17 aislamientos.

Estos dos aislamientos también presentaron motilidad disminuida o nula en el medio Luria Bertani a las 24 horas (figura 3). En el medio mínimo M9, se evidenció una disminución de la motilidad en la mayoría de los aislamientos en estudio; por otra parte, la cepa del aislamiento 1 presentó mayor motilidad comparada con la cepa de control, en tanto que las de los aislamientos 7, 9 y 20 presentaron motilidad disminuida o nula (figura 3).

**Figura 4:**
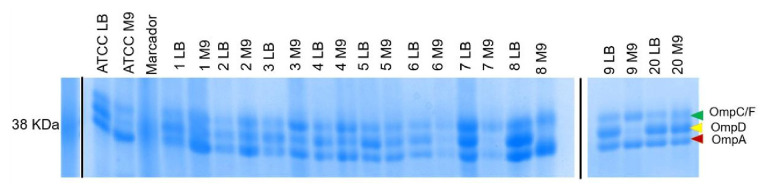
Patrones electroforéticos de preparaciones de proteínas de membrana externa separadas por SDS-PAGE al 10 % y teñidas con azul de Coomassie a partir de los aislamientos que mostraron diferencias en la motilidad (aislamientos 1, 2, 3, 4, 5, 6, 7, 8 y 9) en comparación con la cepa de control ATCC 14028. Se observa una disminución en la expresión de OmpD en el medio mínimo en los aislamientos 7, 8 y 9. Al margen derecho de la imagen, se observa una sección del marcador de peso molecular.

LB: crecimiento en Luria Bertani; M9: crecimiento en medio mínimo M9

No se observó un cambio aparente en la expresión de las principales porinas (OmpC, OmpF, OmpD y OmpA) de las STVM, con excepción de los aislamientos 7, 8 y 9, en los cuales se observó que la porina OmpD desapareció en el medio mínimo M9, y hubo una disminución aparente en la porina OmpA en las muestras 6 y 7 (figura 4).

## Discusión

La variante monofásica de Typhimurium ha circulado a nivel mundial durante las últimas dos décadas ^9^, y representa el tercer serovar más aislado en la Unión Europea, en tanto que en Estados Unidos es de los más frecuentes entre los 20 serovares más comunes ^5^^,^^6^^,^^26^^,^^27^. Este serotipo se identificó por primera vez a finales de los 80 en aves de corral de Portugal ^28^ y, a partir de entonces, se ha convertido en uno de los principales serotipos asociados con la cadena alimentaria porcina, lo que sugiere una transmisión directa de la infección en humanos a partir del consumo de productos contaminados derivados del cerdo en Europa, Estados Unidos y China ^10^^,^^29^^-^^32^.

Además de Estados Unidos, en países del resto de América como Canadá y Brasil, la STVM también se encuentra en los cinco primeros lugares de aislamientos provenientes de muestras de humanos, en tanto que el 100 % de los aislamientos de animales de engorde en estos países corresponde a dicha variante ^33^^,^^34^, En Argentina solo existe un reporte reciente de su presencia en granjas de cerdos ^35^. En Colombia, en solo cinco años desde su primera identificación en aislamientos clínicos, la variante monofásica se ubicó en el cuarto lugar de la vigilancia, lo que evidencia su capacidad de diseminación ^14^. La variante STVM fue confirmada recientemente en aislamientos clínicos recuperados de hemocultivos con los secuenciotipos ST19 y ST34, resistentes a uno o dos antimicrobianos, lo que los diferencia de los clones español y europeo multirresistentes ^15^.

En el presente estudio, se confirmaron los resultados obtenidos previamente sobre la presencia de esta variante en el país, pues el 61 % de los aislamientos amplificaron el marcador para STVM, así como el predominio de los clones europeo-español. La caracterización por PCR y la secuenciación de genoma completo también evidenciaron que el clon estadounidense y el clon endémico ocuparon el segundo lugar, así como la presencia de clones identificados como variantes inconsistentes y atípicas. La detección de esta gran variedad de clones en el país sugiere múltiples fuentes de infección asociadas, probablemente, con las diferentes cadenas pecuarias. Nuestros resultados concuerdan con el panorama mundial de diseminación del clon europeo de la STVM, el cual ha reemplazado a los otros dos ^36^.

Desde el punto de vista epidemiológico, la STVM tiene una estrecha relación con la cadena porcícola, principalmente en Europa y Estados Unidos, lo que sugiere una relación directa entre estos productos alimenticios y las infecciones en humanos. También se recupera de otras fuentes, como el ganado y las aves, lo que demuestra que es una variante con un amplio rango de huéspedes ^9^.

Hasta la fecha en el país, no hay estudios que reporten la presencia de STVM en la cadena porcícola; sin embargo, dado que el consumo de alimentos contaminados es una de las fuentes de la enfermedad diarreica aguda, una hipótesis es que estos aislamientos provienen principalmente de cerdos y están asociados con el clon español, cuyo principal reservorio es el cerdo ^37^. En este sentido, es importante mencionar los múltiples reportes de Typhimurium en comidas rápidas callejeras, en carne de pollo cruda ^38^^,^^39^, carne de cerdo ^40^ y alimentos listos para el consumo humano ^41^, que podrían ser el antecedente para la aparición y rápida diseminación de la STVM, ya que su evolución a partir de aislamientos de Typhimurium ha sido confirmada por varios autores mediante diversas técnicas moleculares y secuenciación de genoma completo en los últimos tiempos ^20^^,^^25^^,^^37^^,^^42^.

Para verificarlo, se requieren estudios de búsqueda de la STVM en granjas, animales o alimentos derivados de cerdos, pues con base en los hallazgos que aquí se presentan, se esperaría una estrecha relación con los aislamientos analizados.

En cuanto a las características fenotípicas de los aislamientos de STVM, por lo general, la ausencia de la segunda fase flagelar no altera el crecimiento ni la motilidad de los aislamientos evaluados. Estos resultados concuerdan con lo reportado por Crayford, *et al.,* quienes observaron que los aislamientos monofásicos conservan la habilidad de adherirse e invadir las células epiteliales del intestino de cerdo *in vitro*^43^.

Sin embargo, algunas particularidades observadas en este estudio llaman la atención: el aislamiento 1, considerado inconsistente, tuvo una motilidad incrementada en el medio mínimo M9 y, aunque contenía los tres genes del operón *fljAB,* no expresó la segunda fase flagelar, lo cual puede deberse a un bloqueo del promotor que controla la expresión de *fljB* y *fliC* o a mutaciones puntuales en estos genes ^12^, en tanto que los aislamientos 7 y 9, provenientes de muestras clínicas de orina, correspondieron a clones diferentes, presentaron motilidad disminuida o nula, así como una disminución en la expresión de OmpD en el medio mínimo M9.

Dados los alcances de este estudio, no se pudo establecer una asociación directa entre las características de los aislamientos y el tipo de muestra o infección del cual fueron recuperados, para lo que se requerirán más estudios. Las infecciones urinarias por *Salmonella* no son frecuentes y este tipo de muestra se puede recuperar por colonización directa de la uretra o por diseminación hematógena a partir del aparato gastrointestinal ^44^. El papel de la porina OmpD en infecciones urinarias por *Salmonella* se desconoce; sin embargo, se ha demostrado que su represión puede ser necesaria para una eficiente proliferación intracelular de *Salmonella*^45^, así como para favorecer la supervivencia dentro del macrófago y aumentar la infección sistémica en modelos en ratón ^46^, lo que sugiere que la represión de OmpD en la STVM podría requerirse en infecciones urinarias. Será necesario hacer otros estudios para aclarar este punto.

Por último, en el estudio se demostró la circulación de STVM en aislamientos clínicos colombianos relacionados con los clones europeo-español y estadounidense, con una posible fuente de transmisión zoonótica y las características fisiológicas descritas para Typhimurium, excepto en dos aislamientos recuperados de muestras de orina. Estos hallazgos sugerirían una relación entre la STVM y su virulencia, lo cual debe confirmarse en futuros estudios.

## References

[1] World Health Organization *Salmonella* (no tifoidea).

[2] Grimont PA, Weill FX (2007). Antigenic formulae of the *Salmonella* serovars.

[3] Yamamoto S, Kutsukake K (2006). Fl/A-mediated posttranscriptional control of phase 1 flagellin expression in flagellar phase variation of Salmonella entérica serovar Typhimurium. J Bacteriol.

[4] Bonifield HR, Hughes KT (2003). Flagellar phase variation in Salmonella enterica is mediated by a posttranscriptional control mechanism. J Bacteriol.

[5] Centers for Disease Control and Prevention (CDC) (2018). National Enteric Disease Surveillance. Salmonella Annual Report 2016.

[6] European Food Safety Authority and European Centre for Disease Prevention and Control (2019). The European Union One Health 2018 Zoonoses Report. EFSA.

[7] Arai N, Sekizuka T, Tamamura Y, Tanaka K, Barco L, Izumiya H (2018). Phylogenetic characterization of Salmonella enterica serovar Typhimurium and its monophasic variant isolated from food animals in japan revealed replacement of major epidemic clones in the last 4 decades. J Clin Microbiol.

[8] Mastrorilli E, Pietrucci D, Barco L, Ammendola S, Petrin S, Longo A (2018). A comparative genomic analysis provides novel insights into the ecological success of the monophasic salmonella serovar 4,[5],12:i:. Front Microbiol.

[9] Sun H, Wan Y, Du P, Bai L (2020). The epidemiology of monophasic Salmonella typhimurium. Foodborne Pathog Dis.

[10] Hauser E, Tietze E, Helmuth R, Junker E, Blank K, Prager R (2010). Pork contaminated with Salmonella enterica serovar 4,[5],12:i:-, an emerging health risk for humans. Appl Environ Microbiol.

[11] Soyer Y, Moreno-Switt A, Davis MA, Maurer J, McDonough PL, Schoonmaker-Bopp DJ (2009). Salmonella enterica serotype 4,5,12:i:-, an emerging Salmonella serotype that represents multiple distinct clones. J Clin Microbiol.

[12] Barco L, Longo A, Lettini AA, Cortini E, Saccardin C, Minorello C (2014). Molecular characterization of "inconsistent" variants of Salmonella Typhimurium isolated in Italy. Foodborne Pathog Dis.

[13] Rodríguez EC, Díaz-Guevara P, Moreno J, Bautista A, Montaño L, Realpe ME (2017). Laboratory surveillance of Salmonella enterica from human clinical cases in Colombia 2005-2011. Enferm Infecc Microbiol Clin.

[14] Instituto Nacional de Salud (2018). Vigilancia por laboratorio de *Salmonella* spp.

[15] Li Y, Pulford CV, Díaz P, Pérez-Sepúlveda BM, Duarte C, Predeus AV (2019). Genomic and phylogenetic analysis of Salmonella Typhimurium and its monophasic variants responsible for invasive endemic infections in Colombia. BioRxiv.

[16] Clinical and Laboratory Standards Institute (2017). Performance standards for antimicrobial susceptibility testing.

[17] Green MR, Michael R, Sambrook J. (2014). Molecular cloning: A laboratory manual.

[18] Pérez-Sepúlveda BM, Heavens D, Pulford CV, Predeus AV, Low R, Webster H (2020). An accessible, efficient and global approach for the large-scale sequencing of bacterial genomes. BioRxiv.

[19] McClelland M, Sanderson KE, Spieth J, Clifton SW, Latreille P, Courtney L (2001). Complete genome sequence of Salmonella enterica serovar Typhimurium LT2. Nature.

[20] Echeita MA, Herrera S, Usera MA (2001). Atypical, flB-negative Salmonella enterica subsp. enterica strain of serovar 4,5,12:i:- appears to be a monophasic variant of serovar Typhimurium. J Clin Microbiol.

[21] Wattam AR, Davis JJ, Assaf R, Boisvert S, Brettin T, Bun C (2017). Improvements to PATRIC, the all-bacterial Bioinformatics Database and Analysis Resource Center. Nucleic Acids Res.

[22] Bogomolnaya LM, Aldrich L, Ragoza Y, Talamantes M, Andrews KD, McClelland M (2014). Identification of novel factors involved in modulating motility of Salmonella enterica serotype typhimurium. PLoS ONE.

[23] Villarreal JM, Becerra-Lobato N, Rebollar-Flores JE, Medina-Aparicio L, Carbajal-Gómez E, Zavala-García ML (2014). The Salmonella enterica serovar Typhi ltrR-ompR-ompC-ompF genes are involved in resistance to the bile salt sodium deoxycholate and in bacterial transformation. Mol Microbiol.

[24] Echeita-Sarrionandia MA, León SH, Baamonde CS (2011). Invasive gastroenteritis, anything new?. Enferm Infecc Microbiol Clin.

[25] Hopkins KL, Kirchner M, Guerra B, Granier SA, Lucarelli C, Porrero MC (2010). Multiresistant Salmonella enterica serovar 4,[5],12:i:- in Europe: A new pandemic strain?. Euro Surveill.

[26] Cito F, Baldinelli F, Calistri P, Di Giannatale E, Scavia G, Orsini M (2016). Outbreak of unusual Salmonella enterica serovar Typhimurium monophasic variant 1,4 [5],12:i:-, Italy, June 2013 to September 2014. Euro Surveill.

[27] European Food Safety Authority, European Centre for Disease Prevention and Control (2017). The European Union summary report on trends and sources of zoonoses, zoonotic agents and food-borne outbreaks in 2016. EFSA J.

[28] Machado J, Bernardo F (1990). Prevalence of Salmonella in chicken carcasses in Portugal. J Appl Bacteriol.

[29] Echeita MA, Aladueña A, Cruchaga S, Usera MA (1999). Emergence and spread of an atypical Salmonella enterica subsp. enterica serotype 4,5,12:i:- strain in Spain. J Clin Microbiol.

[30] Helmuth IG, Espenhain L, Ethelberg S, Jensen T, Kjeldgaard J, Litrup E (2019). An outbreak of monophasic Salmonella Typhimurium associated with raw pork sausage and other pork products, Denmark 2018-19. Epidemiol Infect.

[31] Magossi G, Bai J, Cernicchiaro N, Jones C, Porter E, Trinetta V (2019). Seasonal presence of Salmonella spp., Salmonella Typhimurium and its monophasic variant serotype I 4,[5],12:i:-, in selected United States Swine Feed Mills. Foodborne Pathog Dis.

[32] Yang X, Wu Q, Zhang J, Huang J, Guo W, Cai S (2015). Prevalence and characterization of monophasic Salmonella serovar 1,4,[5],12:i:- of food origin in China. PLoS ONE.

[33] Mulvey MR, Finley R, Allen V, Ang L, Bekal S, El Bailey S (2013). Emergence of multidrug-resistant Salmonella enterica serotype 4,[5],12:i:- involving human cases in Canada: Results from the Canadian Integrated Program on Antimicrobial Resistance Surveillance (CIPARS), 2003-10. J Antimicrob Chemother.

[34] Tavechio AT, Fernandes SA, Ghilardi ÂC, Soule G, Ahmed R, Melles CE (2009). Tracing lineage by phenotypic and genotypic markers in Salmonella enterica subsp. enterica serovar 1,4,[5],12:i:- and Salmonella Typhimurium isolated in state of São Paulo, Brazil. Mem Inst Oswaldo Cruz.

[35] Vico JP, Lorenzutti AM, Zogbi AP, Aleu G, Sánchez IC, Caffer MI (2020). Prevalence, associated risk factors, and antimicrobial resistance profiles of non-typhoidal Salmonella in large scale swine production in Córdoba, Argentina. Res Vet Sci.

[36] Elnekave E, Hong S, Mather AE, Boxrud D, Taylor AJ, Lappi V (2018). Salmonella enterica serotype 4,[5],12:i:- in Swine in the United States Midwest: An emerging multidrug-resistant clade. Clin Infect Dis.

[37] de la Torre E, Zapata D, Tello M, Mejía W, Frías N, García-Peña FJ (2003). Several Salmonella enterica subsp. enterica serotype 4,5,12:i:- phage types isolated from swine samples originate from serotype typhimurium DT U302. J Clin Microbiol.

[38] Donado-Godoy P, Clavijo V, León M, Arévalo A, Castellanos R, Bernal J (2014). Counts, serovars, and antimicrobial resistance phenotypes of Salmonella on raw chicken meat at retail in Colombia. J Food Prot.

[39] Rodríguez JM, Rondón IS, Verjan N (2015). Serotypes of Salmonella in broiler carcasses marketed at Ibagué, Colombia. Rev Bras Cienc Avic.

[40] Rondón-Barragán IS, Arcos EC, Mora-Cardona L, Fandiño C (2015). Characterization of Salmonella species from pork meat in Tolima, Colombia. Revista Colombiana de Ciencias Pecuarias.

[41] Durango J, Arrieta G, Mattar S (2004). Presencia de Salmonella spp. en un área del Caribe colombiano: un riesgo para la salud pública. Biomédica.

[42] Petrovska L, Mather AE, AbuOun M, Branchu P, Harris SR, Connor T (2016). Microevolution of monophasic Salmonella Typhimurium during epidemic, United Kingdom, 2005-2010. Emerging Infect Dis.

[43] Crayford G, Coombes JL, Humphrey TJ, Wigley P (2014). Monophasic expression of FliC gen by Salmonella 4,[5],12:i:- DT193 does not alter its pathogenicity during infection of porcine intestinal epithelial cells. Microbiology.

[44] Tena D, González-Praetorius A, Pérez-Pomata MT, Gimeno C, Alén MJ, Robres P (2000). Urinary infection caused by non typhi Salmonella. Enferm Infecc Microbiol Clin.

[45] Eriksson S, Lucchini S, Thompson A, Rhen M, Hinton JCD (2003). Unravelling the biology of macrophage infection by gene expression profiling of intracellular Salmonella enterica. Mol Microbiol.

[46] Ipinza F, Collao B, Monsalva D, Bustamante VH, Luraschi R, Alegría-Arcos M (2014). Participation of the Salmonella OmpD porin in the infection of RAW264.7 macrophages and BALB/c mice. PLoS ONE.

